# Coupling of Nanocrystalline Anatase TiO_2_ to Porous Nanosized LaFeO_3_ for Efficient Visible-Light Photocatalytic Degradation of Pollutants

**DOI:** 10.3390/nano6010022

**Published:** 2016-01-20

**Authors:** Muhammad Humayun, Zhijun Li, Liqun Sun, Xuliang Zhang, Fazal Raziq, Amir Zada, Yang Qu, Liqiang Jing

**Affiliations:** Key Laboratory of Functional Inorganic Materials Chemistry, Ministry of Education, School of Chemistry and Materials Science, Heilongjiang University, Harbin 150080, China; E-Mails: humayun096@yahoo.com (M.H.); ushlj2008@163.com (Z.L.); sunliqun001@163.com (L.S.); zxlzs007@gmail.com (X.Z.); Rabiabi73@gmail.com (F.R.); amistry009@yahoo.com (A.Z.)

**Keywords:** nanostructures, semiconductors, chemical synthesis, X-ray diffraction, catalytic properties

## Abstract

In this work we have successfully fabricated nanocrystalline anatase TiO_2_/perovskite-type porous nanosized LaFeO_3_ (T/P-LFO) nanocomposites using a simple wet chemical method. It is clearly demonstrated by means of atmosphere-controlled steady-state surface photovoltage spectroscopy (SPS) responses, photoluminescence spectra, and fluorescence spectra related to the formed OH^−^ radical amount that the photogenerated charge carriers in the resultant T/P-LFO nanocomposites with a proper mole ratio percentage of TiO_2_ display much higher separation in comparison to the P-LFO alone. This is highly responsible for the improved visible-light activities of T/P-LFO nanocomposites for photocatalytic degradation of gas-phase acetaldehyde and liquid-phase phenol. This work will provide a feasible route to synthesize visible-light responsive nano-photocatalysts for efficient solar energy utilization.

## 1. Introduction

Photocatalytic materials have received tremendous attention in recent years due to the increase in world-wide environmental pollution. Numerous photocatalysts including nanoparticles of oxides, noble metals, and their nanocomposites have been explored for their superior performance in the degradation of organic pollutants under visible irradiation [1,2]. Recently, considerable attention has been focused on perovskite-type oxides with the general formula ABO_3_, where site A is a rare-earth element and site B is 3D transition metal [3]. In perovskite oxides, the presence of B-site metal cations and oxygen vacancies are of great significance, because the catalytic process mainly depends on the redox properties of B-site metal cations, whereas the oxygen vacancies provide the activation and adsorption sites for the substrates [4]. Hence, it is easy to alter the energy band gap, photogenerated charge separation and then photocatalytic activity [5,6]. It is generally accepted that there is a great potential for ABO_3_-type oxides to be taken as efficient photocatalysts. Among the well-known ABO_3_-type perovskite oxides, LaFeO_3_ (LFO) has attracted much attention owing to its potential applications in photocatalysis, gas sensors, solid-oxide fuel cells, and electronic and magnetic materials [7].

LFO has been chosen as an efficient photocatalyst due its narrow band gap (2.0 eV), which is active under visible light [8]. However, the photocatalytic activity of LFO is still limited, which is attributed to the weak photogenerated charge separation. Similar to other visible-responsive oxide photocatalysts, it has a low conduction band position, located below the standard hydrogen electrode reduction level, which allows fast recombination of photogenerated charge-carriers [9]. Another drawback of traditional LFO is that the materials possess low surface areas [10]. To improve the photocatalytic performance of LFO, elemental doping, coupling with semiconducting metal-oxides and increasing surface areas by introducing pores are widely employed [4,11,12,13]. In general, it is widely accepted that the photogenerated high-energy electrons of narrow band-gap oxides could relax to the bottom of conduction band in extremely short time [2]. This would lead to the fast recombination of photogenerated charges. To overcome this shortfall, the couplings of wide band gap oxides are highly desirable.

In our previous works [2,14], the visible-light activities of BiVO_4_ and Fe_2_O_3_ were obviously improved by coupling TiO_2_ with high-level conduction bottom, primarily demonstrating that this idea is feasible. In such heterojunctions, the visible-light-excited high-energy electrons (from valance band (VB) of narrow band gap oxides) could transfer thermodynamically from the CB of narrow band gap oxides to the other constitution with high energy platform. To the best of our knowledge, there is no previous report on the enhanced visible-light activities of large surface area porous LFO for photocatalytic degradation of colorless organic pollutants by coupling with nanocrystalline anatase TiO_2_.

Herein, we present our work on the visible-light enhanced photocatalytic activities of T/P-LFO nanocomposites for efficient degradation of gas-phase acetaldehyde and liquid-phase phenol. Based on our experimental results, it is suggested that the enhanced photoactivities are attributed to the improved separation of electron-hole pairs in the resulting T/P-LFO nanocomposites. This work will provide feasible routes to synthesize visible-light responsive nano-photocatalysts for efficient solar energy utilization.

## 2. Results and Discussion

### 2.1. Structural Characterization and Surface Composition

**Figure 1:**
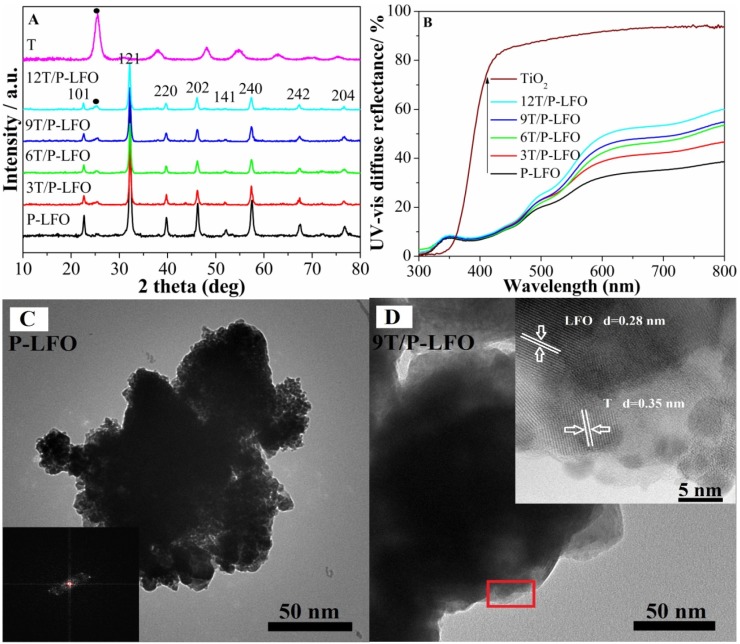
X-ray diffraction (XRD) patterns (**A**) and UV-vis diffuse reflectance (UV-vis DRS) spectra (**B**) of Porous-LaFeO_3_ (P-LFO) TiO_2_ (T) and TiO_2_/P-LFO (T/P-LFO) nanocomposites; Transmission electron microscopy (TEM) image of P-LFO with inset selected area electron diffraction (SAED) pattern (**C**); TEM image of 9T/P-LFO with inset high resolution transmission electron microscopy (HRTEM) image (**D**).

Figure 1A shows the X-ray diffraction (XRD) patterns of P-LFO, TiO_2_ (anatase) and T/P-LFO nanocomposites. The XRD patterns of P-LFO calcined at 600 °C show diffraction peaks at 22.45, 32.13, 39.66, 46.06, 52.01, 57.42, 67.32, 76.63, respectively, correspond to the (101), (121), (220), (202), (141), (240), (242) and (204) reflections of orthorhombic phase LaFeO_3_ (JCPDS No. 37-1493) without any impurity phase. The sharp peaks imply the high crystallinity of the product [15]. In the XRD patterns of T/P-LFO nanocomposites calcined at 450 °C, no impurity peaks can be observed except for TiO_2_ (T) and LFO phases. In addition, the relative peak intensity of TiO_2_ in T/P-LFO nanocomposites is obviously enhanced with an increase in mole ratio percentage of coupled TiO_2_.

The particle size of P-LFO was calculated using the Scherrer formula, which is approximately 50 nm. According to the UV-vis DRS spectra (Figure 1B), the absorption lower than 400 nm for TiO_2_ is attributed to the electronic transitions from its valance band to the conduction band (O2p → La3d). While the absorption edge of P-LFO appeared at approximately 620 nm, attributed to its electronic transition from the valance band to conduction band (O2p → Fe3d), and the optical absorption across the energy band gap is 2.0 eV.

To investigate the morphology and microstructure of P-LFO and 9T/P-LFO nanocomposite, the TEM and HRTEM micrographs were taken. One can see that the as-synthesized P-LFO exhibits random distribution with a crystallite size of about 50 nm determined from the TEM micrograph (Figure 1C). This is consistent with the crystallite size calculated from the XRD patterns. Moreover, the nanocrystalline orthorhombic-phase structure of P-LFO is confirmed from the selected area electron diffraction (SAED) patterns (inset of Figure 1C). From the HRTEM micrograph of the 9T/P-LFO nanocomposite (Figure 1D inset), it can be seen that an intimate interface junction exists between TiO_2_ and P-LFO. The lattice fringes at (121) plane with d-spacing 0.28 nm correspond to the orthorhombic phase LFO [16], while the latter at (101) plane with d-spacing 0.35 nm are attributed to the anatase TiO_2_ [17].

**Figure 2:**
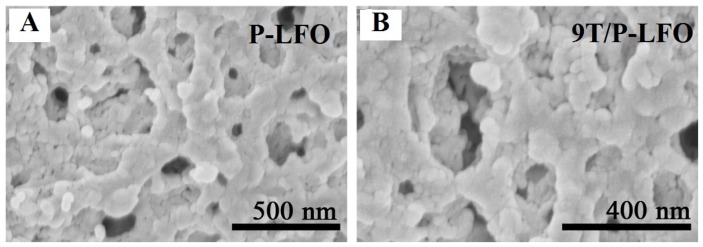
Scanning electron microscopy (SEM) micrograph of P-LFO (**A**) and 9T/P-LFO nanocomposite (**B**).

Figure 2A shows the SEM micrograph of P-LFO calcined at 600 °C. It can be seen clearly, that the P-LFO exhibits an irregular porous morphology. Moreover, it is suggested that the P-LFO nanoparticles are inter-connected and a large network of irregular shapes and sizes are formed due to the escape of a large number of gases due to the strong redox reaction taking place during the sol-gel auto-combustion. After coupling with nanocrystalline anatase TiO_2_, the morphology of the nanoparticles are slightly changed as shown in Figure 2B. The XRD, TEM and SEM results demonstrate that TiO_2_/P-LFO nanocomposites were successfully fabricated.

**Figure 3:**
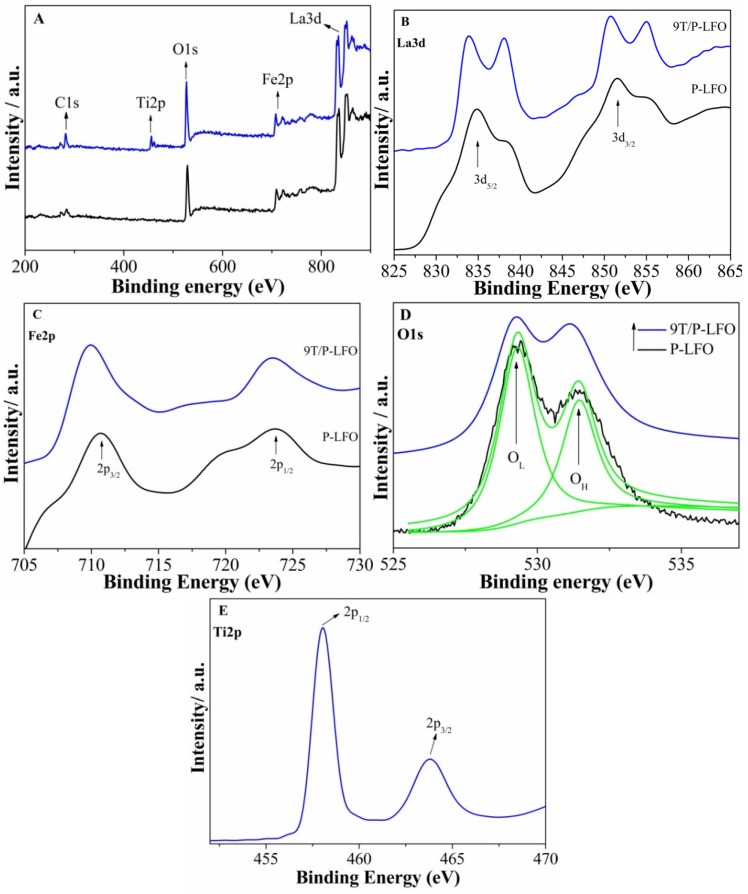
X-ray photoelectron spectroscopy (XPS) survey spectra of P-LFO and 9T/P-LFO nanocomposite (**A**); with high resolution images La3d (**B**); Fe2p (**C**); O1s (**D**); Ti2p (**E**).

The surface functional groups and elemental states of the P-LFO and 9T/P-LFO nanocomposite was characterized by X-ray photoelectron spectroscopy (XPS). The binding energies were calibrated with respect to the adventitious carbon (C1s) as a reference line at 284.6 eV. The typical survey spectra (Figure 3A), of P-LFO and 9T/P-LFO samples reveals the presence of La3d, Fe2p, O1s, Ti2p and C1s. In the high-resolution spectrum of La3d for P-LFO (Figure 3B), two intense peaks, observed at 833.6 and 850.5 eV respectively, correspond to the spin-orbital splitting of 3d_5/2_ and 3d_3/2_ of La^3+^ ions in the oxide form. It can be observed that after coupling with TiO_2_, the binding energies of La3d for 9T/P-LFO nanocomposite are slightly shifted toward lower binding energies. For Fe2p (Figure 3C), the binding energies observed at 710 (Fe2p_3/2_) and 723.6 eV (Fe2p_1/2_) respectively, correspond to the +3 oxidation state of Fe in P-LFO oxide. In addition, the binding energies of Fe2p for 9T/P-LFO nanocomposite are also shifted toward lower energies. The XPS spectra of O1s (Figure 3D) are fitted into two separate peaks with origin software by the Gaussian rule. The XPS spectra of O1s are broad and asymmetric, demonstrating that there exist two kinds of O chemical states, including crystal lattice oxygen (OL) and hydroxyl oxygen (OH) with increasing binding energy. The XPS signal for OL corresponds to the La-O and Fe-O in P-LFO crystal lattice and appeared at approximately 529.5 eV, while the OH XPS signal lies at about 531.5 eV, and is closely related to the hydroxyl species resulting from the chemisorbed water [18,19]. Besides, the binding energy of Ti2p (Figure 3E) at 458 eV corresponds to TiO_2_ in 9T/P-LFO nanocomposite [20]. Hence, it is suggested that an intimate contact exist between TiO_2_ and P-LFO interfaces. Obviously, this is in good agreement with the TEM results.

**Figure 4:**
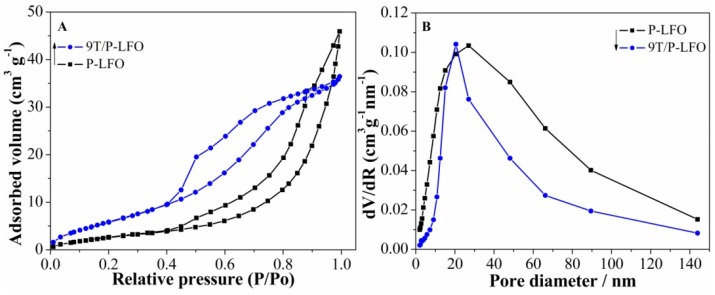
N_2_ adsorption/desorption isotherms (**A**) and pore diameter (**B**) of P-LFO and 9T/P-LFO nanocomposite.

The nitrogen adsorption-desorption isotherms and the corresponding pore size distribution of P-LFO and 9T/P-LFO nanocomposite respectively, are depicted in Figure 4A,B. It can be seen clearly that the sorption isotherms of P-LFO and 9T/P-LFO exhibit hysteresis loops, which are the characteristics of porous structure [16]. The Brunauer-Emmett-Teller (BET) surface area observed for P-LFO is 23.2 m^2^·g^−1^ and its BJH average pore diameter is 7.4585 nm, while for 9T/P-LFO nanocomposite, the BET surface area is considerably higher (34.2 m^2^·g^−1^) as compared to P-LFO. This is well attributed to the small size with large surface area of coupled TiO_2_ [21].

### 2.2. Photogenerated Charge Properties

**Figure 5:**
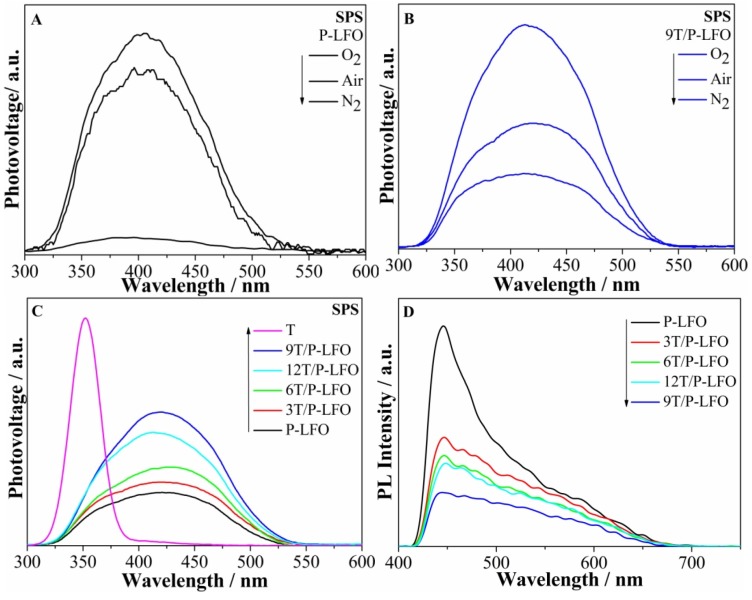
Surface photovoltage spectroscopy (SPS) responses of P-LFO (**A**) and 9T/P-LFO (**B**) in different atmospheres; SPS responses of P-LFO and T/P-LFO nanocomposites in air (**C**); Photoluminescence (PL) responses of P-LFO and T/P-LFO nanocomposites (**D**).

Surface photovoltage spectroscopy (SPS) is a highly sensitive and non-destructive technique used to study the photophysics of the photogenerated charges in semiconducting solid materials, resulting from the changes of surface potential barriers before and after illumination. The SPS response for nanocrystalline semiconductors would mainly be derived from photo-generated charge separation via the diffusion process. From the SPS responses of P-LFO in different atmospheres in Figure 5A, it can be seen that the SPS response intensity is greatly influenced by the amount of O_2_. In N_2_ atmosphere, no obvious SPS response is detected for P-LFO, suggesting that the presence of O_2_ is necessary for SPS response to occur. This supports the role of adsorbed O_2_ in capturing the photogenerated electrons. However, for 9T/P-LFO nanocomposite Figure 5B, a remarkable SPS response is detected in N_2_ atmosphere, although its SPS response is also enhanced with the increase in O_2_ concentration. This unexpected SPS response in N_2_ is mainly attributed to the improved separation of photogenerated charges in the fabricated T/P-LFO nanocomposites [2]. From Figure 5C, it can be seen clearly that the SPS response of P-LFO in air atmosphere is obviously increased after coupling with TiO_2_ and the strongest response is observed for 9T/P-LFO nanocomposite, suggesting that the charge transfer and separation is significantly improved [9,22]. However, the excess amount of TiO_2_ is unfavorable for the charge transfer and separation. From SPS measurements, it is confirmed that T/P-LFO nanocomposites could exhibit high photoactivities.

The photoluminescence (PL) is a highly sensitive and non-destructive technique widely used to investigate the structure and properties of active sites on the surface of metal oxides. It always gives us information about the surface defects and oxygen vacancies, as well as about charge carrier trapping, immigration and transfer [22]. The PL spectra of P-LFO and T/P-LFO nanocomposites under excitation wavelength of 325 nm are depicted in (Figure 5D). It can be observed that the PL peak intensity of P-LFO is considerably decreased after coupling with nanocrystalline TiO_2_, and the lowest PL response is detected for 9T/P-LFO nanocomposite, suggesting that the charge recombination is significantly reduced. These results are in good agreement with the above SPS results.

### 2.3. Visible-Light Photoactivities

**Figure 6:**
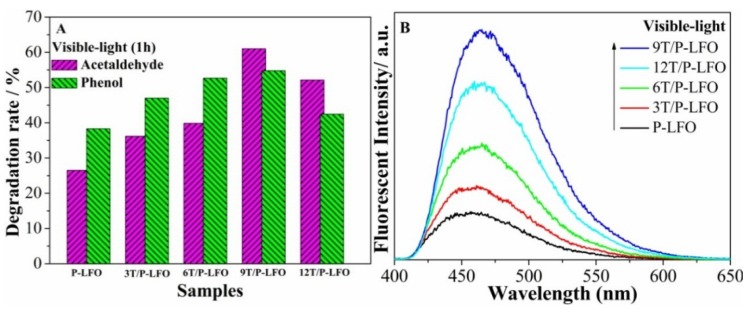
Visible-light photocatalytic activity for acetaldehyde and phenol degradation (**A**) and OH^−^ radical amount related Fluorescence spectra (**B**) of P-LFO and T/P-LFO nanocomposites.

To evaluate the visible-light activities of P-LFO and T/P-LFO nanocomposites for pollutant degradation, gas-phase acetaldehyde and liquid-phase phenol were chosen as model pollutants. The visible-light photocatalytic activity of P-LFO and T/P-LFO nanocomposites for acetaldehyde and phenol degradation is depicted in Figure 6A. It can clearly be seen that the photocatalytic degradation rates of P-LFO for acetaldehyde and phenol are greatly enhanced after coupling with TiO_2_. Interestingly, 9T/P-LFO exhibits the highest photocatalytic activity. Hence, it is suggested that coupling a proper mole ratio percentage of TiO_2_ is favorable for charge transfer and separation, which further leads to the enhanced visible-light activities [14].

### 2.4. Discussion

To prove the visible-light enhanced charge transfer and separation in the fabricated T/P-LFO nanocomposites, the coumarin fluorescent method was used to detect the amount of formed (•OH) species. As illustrated in our previous reported work [23], the amount of (•OH) species could also effectively reveal the separation of photogenerated charges in photocatalysis. It is demonstrated that in coumarin fluorescent process, the coumarin could easily react with the formed •OH species and produce luminescent 7-hydroxy-coumarin. From (Figure 6B), it can be observed that the fluorescent response intensity of P-LFO is obviously enhanced after coupling with TiO_2_ and 9T/P-LFO nanocomposite exhibit the strongest fluorescent intensity peak. This further supports the SPS results and photoactivities.

**Figure 7:**
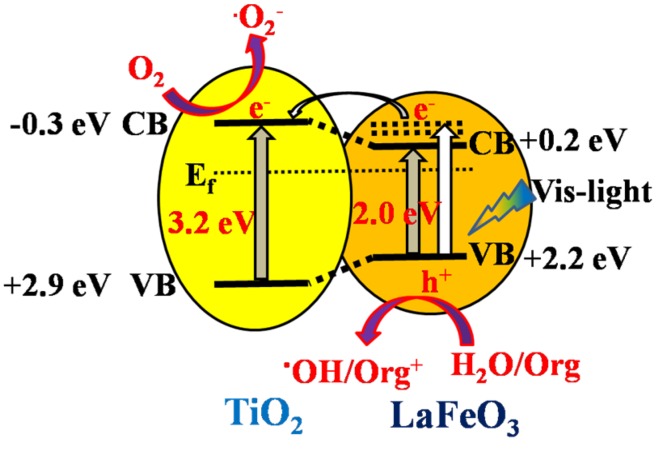
Scheme for energy band gaps and the mechanism for photogenerated charge separation and transfer in the fabricated T/P-LFO nanocomposite.

Based on the above results and discussion, a possible mechanism for charge transfer and separation in T/P-LFO nanocomposites is proposed as depicted in Figure 7. It is suggested that when T/P-LFO nanocomposite is radiated by visible-light with photon energy higher than the band gap of P-LFO, electron-hole pairs are produced. The high-energy electrons would transfer thermodynamically to the CB of TiO_2_, which would probably react with the surface adsorbed O_2_ to produce superoxide radicals •O_2_**^−^**, while the holes will remain in the VB of P-LFO and react with the surface adsorbed water or OH^−^ to produce **^.^**OH radicals. In this way the photogenerated charge recombination could be effectively reduced and utilized in the photocatalytic process to enhance the visible-light photoactivities.

## 3. Experimental Section

### 3.1. Chemicals and Reagents

All the reagents were of analytical grade and used as received without further purification. Deionized water was used throughout the experiment.

### 3.2. Synthesis of Porous LaFeO_3_

Porous LaFeO_3_ nanoparticles were synthesized by taking equimolar amounts (0.04 mol) of La(NO_3_)·6H_2_O and Fe(NO_3_)_3_·9H_2_O and dissolved into a mixed solvent of ethylene glycol (EG) and methanol (3:7 vol %) at room temperature. The solution was treated by ultrasonication for 1 h. Then polystyrene (PS) colloidal crystals were soaked in the precursor solution and kept under vigorous magnetic stirring for 12 h. After that, the mixture was dried in oven at 80 °C for 12 h. The dry powder was then calcined in air at 400 °C (temp ramp 1 °C·min^−1^) for 2 h to remove the polystyrene spheres. Finally the product was calcined at 600 °C (5 °C·min^−1^) for 2 h to obtain porous LaFeO_3_ nanoparticles.

### 3.3. Fabrication of Nanocomposite Materials

To fabricate different T/P-LFO nanocomposites, for each sample 1 g freshly prepared P-LFO nanoparticles were taken and suspended into a mixed solvent containing 10 mL water, 40 mL anhydrous ethanol, and 2 mL HNO_3_ (68%), under vigorous stirring at room temperature. The reaction mixtures were treated by ultrasonication for 10 min. Then the mixtures were kept under vigorous stirring for 30 min. After that, a certain volume (0.3, 0.6, 0.9, 1.2 mL) of the mixed solution of Titanium butoxide Ti[(OCH_2_)_3_CH_3_]_4_ and anhydrous ethanol with a ratio of (1:9 vol %) was added to the reaction mixtures under vigorous stirring for 2 h. Subsequently, the mixtures were dried at 85 °C in an oven, followed by calcining at 450 °C for 2 h. Different mole ratios of T/P-LFO nanocomposites were obtained and denoted by *X* T-LF, where *X* represents the mole ratio percentage of Ti to La.

### 3.4. Evaluation of Photocatalytic Activity for Pollutant Degradation

Here in this work, liquid-phase phenol and gas-phase acetaldehyde have been chosen as model pollutants because phenol is a typical recalcitrant contaminant without sensitizing as a dye and acetaldehyde is a kind of volatile organic compound which widely exist in industrial production and are harmful to both human health and natural environment. Therefore, both the pollutants were selected to evaluate the visible-light photocatalytic activities of the fabricated T/P-LFO nanocomposites. The liquid-phase photocatalytic experiments were carried out in an open photochemical reactor glass with 100 mL volume under visible irradiation with cutoff 420 nm wavelength filter using a source of 150 W GYZ220 high-pressure Xenon lamp made in China. The distance of the light source from the reactor glass was approximately 10 cm. In a typical experiment, 0.1 g of photocatalyst and 80 mL of 10 mg/L phenol solution were mixed under stirring for 0.5 h, so as to reach the adsorption saturation and then irradiated under visible-light for 1 h. After irradiation for 1 h, the solution was centrifuged and the concentration of phenol was analyzed with a Model Shimadzu UV-2550 Spectrophotometer (Kyoto, Japan) using colorimetric method of 4-aminoantipyrine at the characteristic optical adsorption of 510 nm.

For the photocatalytic degradation of gas-phase acetaldehyde, the experiments were carried out in a 3 mouth cylindrical quartz reactor with 640 mL volume. A desired amount of photocatalyst and a specific concentration of acetaldehyde gas is introduced through these mouths. After carefully sealed, the reactor was irradiated under visible-light using a source of 150 W Xenon lamp with cutoff filter (λ > 420 nm). In a typical experiment, 0.1 g of photocatalyst was suspended in a quartz reactor containing a mixture of 810 ppm acetaldehyde, 20% O_2_ and 80% of N_2_. Prior to irradiation, the gases were mixed by continuous flow through the reactor for half an hour to reach the adsorption saturation. The concentration of acetaldehyde in the photocatalysis was detected with a gas chromatograph (GC-2014, Shimadzu, Kyoto, Japan) equipped with a flame ionization detector. After that, the sample was irradiated by visible-light for 1 h and the concentration of the acetaldehyde gas was re-measured.

### 3.5. Measurement of the Produced Hydroxyl Radical (•OH) Amount

To measure the amount of hydroxyl radicals, in a typical experiment, 50 mg of photocatalyst and 20 mL of 5 mg·L^−1^ aqueous solution of coumarin were mixed in a 50 mL of quartz glass reactor. The reactor was irradiated under visible-light using a source of 150 W high-pressure Xenon lamp with cutoff filter (λ > 420 nm) under continuous magnetic stirring for 1 h. The distance of the source from the reactor was about 10 cm. After irradiation for 1 h, a certain amount of solution was taken in a Pyrex glass cell for the fluorescence measurement of 7-hydroxycoumarin under excitation wavelength of 350 nm.

### 3.6. Characterization

The materials were characterized by using various techniques. The crystal structures of the samples were determined with the help of XRD (Rigaku D/MAX-RA diffractometer, Kyoto, Japan), operated at an accelerating voltage of 30 kV, using Cu Kα radiation (α = 0.15418 nm). During measurement, the emission current of 20 mA was employed. The UV-vis diffuse reflectance spectra of the samples were obtained with the help of Shimadzu UV-2550 spectrophotometer (Kyoto, Japan), using BaSO_4_ as a reference. Transmission electron microscopy (TEM) micrographs of the samples were taken by a JEOL, JEM-2100 (Tokyo, Japan), electron microscope operated at an acceleration voltage of 300 kV. Scanning electron microscopy (SEM) images were taken using a Hitachi S-4800 instrument (Tokyo, Japan), operating at acceleration voltage of 15 kV. The chemical compositions and elemental states of the samples were tested with the help of Kratos-Axis Ultra DLD X-ray photoelectron spectroscopy (XPS) (Kyoto, Japan), with an Al (mono) X-ray source and the binding energies of the samples were calibrated with respect to the signal for adventitious carbon (binding energy = 284.6 eV). The N_2_ adsorption–desorption isotherm of various samples were carried out by Micromeritics Tristar II 3020 system (Atlanta, GA, USA) at the temperature of liquid nitrogen, while keeping the system out-gassed for 10 h at 150 °C prior to measurements. The photo luminescence (PL) spectra of the samples were measured with a PE LS 55 spectrofluorophotometer (Waltham, MA, USA) at excitation wavelength of 325 nm.

The atmosphere-controlled surface photovoltage spectroscopy (SPS) spectra of the samples were detected with a home-built apparatus, equipped with a lock-in amplifier (SR830, Sunnyvale, CA, USA) synchronized with a light chopper (SR540, Sunnyvale, CA, USA). The powder samples were sandwiched between two indium tin oxide (ITO) glass electrodes, which were fixed in an atmosphere-controlled system with a quartz window. The monochromatic light was obtained by passing light from a source of 500W Xenon lamp (CHF XQ500W, Global xenon lamp power) through a double prism monochromator (SBP3000, Beijing, China).

## 4. Conclusions

We have developed T/P-LFO nanocomposites, which exhibit superior visible-light photocatalytic activity for acetaldehyde and phenol degradation. The enhanced photocatalytic activities of T/P-LFO nanocomposites can be attributed to the effective electron-hole pair separation by transferring electrons from P-LFO to TiO_2_. This research demonstrates that T/P-LFO nanocomposites show promising applications in the field of photocatalysis by utilizing solar energy.
